# P-1977. Adverse Event Profile of Vancomycin vs. Linezolid in Real-World Reports: A Disproportionality Analysis from the AERS Database

**DOI:** 10.1093/ofid/ofaf695.2144

**Published:** 2026-01-11

**Authors:** Ashin Siby, Manu Mathew, Jose T John

**Affiliations:** Durdans Hospital, Colombo, Western Province, Sri Lanka; Durdans Hospital, Colombo, Western Province, Sri Lanka; Durdans Hospital, Colombo, Western Province, Sri Lanka

## Abstract

**Background:**

Vancomycin and linezolid are critical agents in the treatment of multidrug-resistant gram-positive infections, including MRSA and VRE. Despite their effectiveness, both drugs have been associated with distinct adverse event (AE) profiles. This study aimed to compare the post-marketing safety signals of vancomycin and linezolid using real-world data from the U.S. FDA Adverse Event Reporting System (AERS/FAERS).

**Figure:**
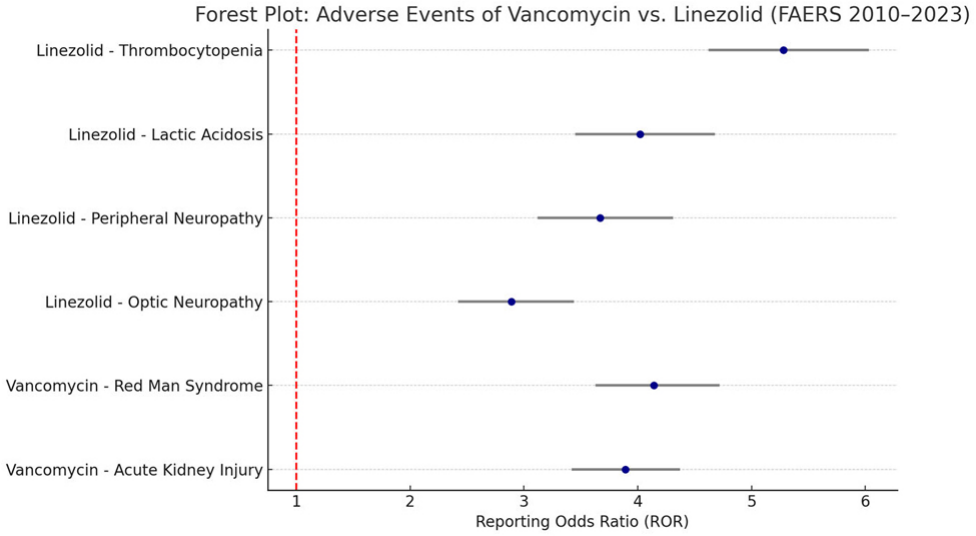
Forest Plot: Adverse Events of Vancomycin vs. Linezolid (FAERS 2010–2023) This forest plot presents the Reporting Odds Ratios (RORs) and 95% confidence intervals for selected adverse events associated with vancomycin and linezolid. Vancomycin showed strong associations with acute kidney injury and Red Man Syndrome, while linezolid demonstrated higher risks for thrombocytopenia, lactic acidosis, peripheral neuropathy, and optic neuropathy. These real-world findings support tailored risk assessment in antibiotic selection and monitoring.

**Methods:**

A retrospective disproportionality analysis was conducted using FAERS data from January 2010 to December 2023. Reports that listed vancomycin or linezolid as the primary suspect drug were included. Adverse events were grouped according to MedDRA System Organ Classes (SOCs) and high-level terms such as nephrotoxicity, thrombocytopenia, lactic acidosis, peripheral neuropathy, and optic neuropathy. Reporting Odds Ratios (RORs) with 95% confidence intervals (CIs) were calculated. A signal was considered significant if the lower bound of the 95% CI was >1 with ≥3 reports.

**Results:**

A total of 19,276 AE reports were included (vancomycin: 11,421; linezolid: 7,855). Vancomycin showed a strong signal for acute kidney injury (ROR: 3.89, 95% CI: 3.42–4.37), and infusion-related reactions including Red Man Syndrome (ROR: 4.14, 95% CI: 3.63–4.72). In contrast, linezolid exhibited higher signals for thrombocytopenia (ROR: 5.28, 95% CI: 4.62–6.03), lactic acidosis (ROR: 4.02), peripheral neuropathy (ROR: 3.67), and optic neuropathy (ROR: 2.89). Mortality was reported in 7.8% of linezolid-associated reports and 6.1% of vancomycin reports.

**Conclusion:**

This FAERS-based analysis reveals distinct and clinically relevant AE profiles for vancomycin and linezolid. Vancomycin is more strongly associated with nephrotoxicity and infusion reactions, whereas linezolid poses a higher risk for hematologic and neurologic toxicity. These findings highlight the importance of individualized risk assessment and monitoring strategies when using these agents in clinical practice.

**Disclosures:**

All Authors: No reported disclosures

